# Supplementary material for: Is birth out-of-hospital associated with mortality and morbidity by seven years of age?
Source: PLoS One. 2021 Apr 21;16(4):e0250163. doi: 10.1371/journal.pone.0250163 (PMC8059817; doi:10.1371/journal.pone.0250163)
Supplement: Attachment — Submitted filename: Review_PONE-D-20-37796.doc [file pone.0250163.s001.doc]

**Review PONE-D-20-37796 Is birth out-of-hospital associated with mortality and morbidity by 7 years of age?**

The manuscript PONE-D-20-37796 presents results from a cohort of 790 136 children, born in Finland between 1996 and 2013. The primary research question was whether place of birth (in-hospital or out-of-hospital) influenced mortality and morbidity risk. As data on longterm mortality and morbidity risk of children, born inside and outside hospital were rare, the results are interesting.

Although it is a huge data set, the study suffers from the surprisingly low proportion of out-of-hospital (OH) births. Only 1514 (0,2%) children were born OH. The authors do not provide any explanation as to why the proportion of OH deliveries is so low in Finland compared to other countries. Because of the small OH proportion, the main research question (place of birth and mortality) cannot be examined conclusively The only valid conclusion is that out-of-hospital births obviously do not increase the mortality risk. The small proportion of out-of-hospital births further limits the question on morbidity considerably, since the observed incident diseases were relatively rare, especially for the group of planned out-of-hospital (POH) births.

Besides the low number of cases in the OH group, for which the authors cannot be held responsible, my main criticisms of the manuscript is the poor description of the methodology and presentation of results.

In detail I have the following comments:

- general remarks:
  - You use too many abbreviations, sometimes with the same content (e.g. UOH and UOHD). This hinders the reading of the manuscript.
- Abstract:
  - The abstract should contain the main results, quantitatively presented as effect sizes with confidence intervals
  - Avoid repetition. The statement that “birth out-of-hospital was not associated with childhood mortality” similar appears twice in the Abstract.
- Methods:
  - Please explain why you are excluding approximately 25% of the original population from the analysis.
  - Apgar score is not mentioned in the methods section but surprisingly appears for the first time in the results section
  - The reasons for planned and unplanned births outside the hospital are so different that the construction of a combined group seems questionable. I would recommend ananlyzing the groups separately only.
- Statistical analyses:
  - I would recommend to omit the statistical tests (U-test, Chi-square-test) and p-values. Statistical tests are only useful in case of predefined hypothesis and a prior sample size calculation. (Please note the current discussion on p-values in the academic literature, for example: Amrhein, V., Greenland, S. & McShane, B. Scientists rise up against statistical significance. Nature 567, 305-307, doi:10.1038/d41586-019-00857-9 (2019)).
  - Regarding regression models, you should always outline how you formed these models, i.e., which is the dependent variable, which is the exposure variable, and which covariates and interaction terms are in the model? Regarding interaction – what significance level did you use to include interaction terms? What is the rationale to analyze interaction?
- Results:
  - In general, the description of the results in the text is poor. Please describe and interpret the main information of the tables in the text quantitatively.
  - All tables: Please attach legends. The legends should explain the abbreviations used. The composition of the sample should be described in the methods section and not again in the legend.
  - Table 1 and 2: In tables 1 and 2, only the sample should be presented descriptively. Why are logistic models used here? These do not serve to answer the primary research question but overload the tables and hinder readability. I would recommend to delete the results of the logistic models.
  - Table 1: Length of gestation at birth: Please describe the rows clearly (22+0-31+6 ???)
  - Table 3-5: As written above, you should avoid the p-values. I would recommend means, relative frequencies, and the respective 95% confidence intervals.
  - Table 6: Why for asthma the column with interaction is empty? What do the asterisk mean? What are the covariates in the multivariate models? Please list the covariates in the legend. Merely writing, “something was higher” or is “statistically significant” without mention the effect size is not enough.
  - Regading neurological or mental disorders, you state that “the analysis with

interactions suggested that birth weight SGA or LGA was associated with increased HRs for later neurological and mental disorders”. However, this is not your research question. Furthermore, in table 6 I see only an effect for SGA (HR = 1,59) but not for LGA (HR = 1,0)…
