# Supplementary material for: Is birth out-of-hospital associated with mortality and morbidity by seven years of age?
Source: PLoS One. 2021 Apr 21;16(4):e0250163. doi: 10.1371/journal.pone.0250163 (PMC8059817; doi:10.1371/journal.pone.0250163)
Supplement: Attachment — Submitted filename: Response_to_Reviewers.docx [file pone.0250163.s002.docx]

Editorial Office

PLOS ONE	Feb 12, 2021

Please find attached our revised manuscript entitled “Is birth out-of-hospital associated with mortality and morbidity by 7 years of age?” to be considered for publication in PLOS ONE. We appreciate the interest that the editors and reviewers have taken in our manuscript and the constructive criticism and feedback they have given. These changes have clearly improved our manuscript and we would like to thank the reviewers for the constructive criticism and feedback.

Reviewer #1 Comments to the Author:


Although it is a huge data set, the study suffers from the surprisingly low proportion of out-of-hospital (OH) births. Only 1514 (0,2%) children were born OH. The authors do not provide any explanation as to why the proportion of OH deliveries is so low in Finland compared to other countries. Because of the small OH proportion, the main research question (place of birth and mortality) cannot be examined conclusively The only valid conclusion is that out-of-hospital births obviously do not increase the mortality risk. The small proportion of out-of-hospital births further limits the question on morbidity considerably, since the observed incident diseases were relatively rare, especially for the group of planned out-of-hospital (POH) births.

We agree that the proportion of children who were born out-of-hospital is low, indicating that the statistical significances may be difficult to show, and this is why the results of our study need to be interpreted with caution. We admit that this is a weakness, as commented in the Discussion. Our data collected from national register is on the other hand reliable and collected from the long study period. The place of birth is registered for each birth and double-checked in unclear situations.

Besides the low number of cases in the OH group, for which the authors cannot be held responsible, my main criticisms of the manuscript is the poor description of the methodology and presentation of results.

We have revised the manuscript, in order to improve the presentation of the methods and results.

In detail I have the following comments:

- general remarks:
  - You use too many abbreviations, sometimes with the same content (e.g. UOH and UOHD). This hinders the reading of the manuscript.

We thank the reviewer for the feedback and we have simplified the use of abbreviations.

- Abstract:
  - The abstract should contain the main results, quantitatively presented as effect sizes with confidence intervals

Hazard ratios with confidence intervals have been included in revised version of the Abstract.

- Avoid repetition. The statement that “birth out-of-hospital was not associated with childhood mortality” similar appears twice in the Abstract.

This has been corrected in the Abstract.

- Methods:
  - Please explain why you are excluding approximately 25% of the original population from the analysis.

We have clarified this in revised version. The reason is avoiding overlapping with our previous reports.

- Apgar score is not mentioned in the methods section but surprisingly appears for the first time in the results section

Thank you very much for detecting of the error. This has been corrected in the Methods.

- The reasons for planned and unplanned births outside the hospital are so different that the construction of a combined group seems questionable. I would recommend analyzing the groups separately only.

We have omitted combined data from the Tables 1 and 2, containing data on characteristics of the mothers and children. However, the children born out-of-hospital have also some clinical features in common, i.e. being not born in the delivery room environment and lack of exposure to intra partum antibiotics, as explained in the Discussion. This is why we decided to also combine these groups in some of the analyses.

- Statistical analyses:
  - I would recommend to omit the statistical tests (U-test, Chi-square-test) and p-values. Statistical tests are only useful in case of predefined hypothesis and a prior sample size calculation. (Please note the current discussion on p-values in the academic literature, for example: Amrhein, V., Greenland, S. & McShane, B. Scientists rise up against statistical significance. Nature 567, 305-307, doi:10.1038/d41586-019-00857-9 (2019)).

This is a very good comment. A revision is made, as requested.

- Regarding regression models, you should always outline how you formed these models, i.e., which is the dependent variable, which is the exposure variable, and which covariates and interaction terms are in the model? Regarding interaction – what significance level did you use to include interaction terms? What is the rationale to analyze interaction?

The statistical methods have been explained more thoroughly.

- Results:
  - In general, the description of the results in the text is poor. Please describe and interpret the main information of the tables in the text quantitatively.

The results have been written more informatively.

- All tables: Please attach legends. The legends should explain the abbreviations used. The composition of the sample should be described in the methods section and not again in the legend.
- Table 1 and 2: In tables 1 and 2, only the sample should be presented descriptively. Why are logistic models used here? These do not serve to answer the primary research question but overload the tables and hinder readability. I would recommend to delete the results of the logistic models.
- Table 1: Length of gestation at birth: Please describe the rows clearly (22+0-31+6???)

We have revised these above mentioned things in tables 1 and 2.

- Table 3-5: As written above, you should avoid the p-values. I would recommend means, relative frequencies, and the respective 95% confidence intervals.

The p-values have been omitted.

- Table 6: Why for asthma the column with interaction is empty? What do the asterisk mean? What are the covariates in the multivariate models? Please list the covariates in the legend. Merely writing, “something was higher” or is “statistically significant” without mention the effect size is not enough.

The numbers into the asthma column with interaction have been added.

- Regading neurological or mental disorders, you state that “the analysis with

interactions suggested that birth weight SGA or LGA was associated with increased HRs for later neurological and mental disorders”. However, this is not your research question. Furthermore, in table 6 I see only an effect for SGA (HR = 1,59) but not for LGA (HR = 1,0

The comment regarding our research question is very correct. The data on SGA, LGA and AGA is only confusing and it has been left out from the Table.

We have modified the Result section according to the above mentioned suggestions of reviewer.

Reviewer #2 Comments to the Author

The authors of this paper describe the impact of the morbidity and mortality of children at age seven, born out-of-hospital (UOH) as compared to those born in-hospital. The study period is almost two decades including almost 800 thousand deliveries in Finland during that period. A strong study on the possible risk of giving birth at home is important. At the same time, the responsibilities in analysing and presenting the data is great. From that point of view, this paper is important and at the same time the demands for a clear analysis and discussion are high. Although the study describes interesting data from a whole country, there are issues that need to be better clarified in the manuscript. I urge the authors to better address several points, especially in the discussion chapter.

The main conclusion of the authors is “The risk for morbidity related to asthma or allergic diseases and infections by 7 years of age was decreased in children born out-of-hospital. Birth out-of-hospital was not associated with increased risk for neurological morbidity and early childhood mortality.” This is a really strong claim, possibly too strong for the results presented in the paper. The authors even say that “….. our study groups were small, indicating that statistical significances may be difficult to show. Thus, we realize that our results need to be interpreted with caution.” I urge the authors to implement this caution in their conclusion.

This is a very good comment. We have written the conclusions into a more cautious form.

An important limitation to this study is in my mind the possible (likely?) selection bias. Parents who choose to have the baby borne at home do certainly have a different background as mentioned in the paper and as can be seen in the tables. These include older mothers, more often multipara, upper socioeconomic class, less smoking etc. Moreover, and quite important, all but one baby in the planned out of-hospital (POH) group were delivered at term. In my mind, these are important differences and may certainly influence the results. The authors may want to add a substantial discussion on this to their paper, addressing clearly these differences and a possible selection bias.

The risk of selection bias is true, indeed. We have added this aspect into the discussion.

Another important point for discussion or explanation is the number of children born UOH. The total number of children born in Finland during the study period was more than one million – certainly a respectable number! Of those almost 800 thousand were included in the study; 788.622 born in-hospital and 176 planned out-of-hospital (POH). Despite the large numbers, the total number of 176 POH over a period of almost two decades is not very high. It reflects approximately 10 children per year and the number of POH to in-hospital deliveries is approximately 1:5.000. This is alluded to in the section on limitations, nevertheless, I find that the authors draw quite strong conclusions from these numbers. These conclusions also partly contradict their conclusions in their earlier publications.

The conclusions have been drawn more cautiously, as suggested. However, we do not agree that our report might contradict the conclusions in our earlier publications, because the present study is focused on the long-term outcome of children born out-of-hospital.

Following are several remarks, both minor as well as major that the authors may want to allude to.

Probably the group of unplanned out-of-hospital deliveries (UOH) is a very different group as compared to planned out of-hospital (POH) deliveries. The authors chose to combine these two groups in some instances. That may very well be interesting, but I find it a little confusing.

Please see the reply to Reviewer 1.

In the results chapter in the abstract no numbers/percentages/statistical results are mentioned.

More details on statistical results have been added into the Abstract.

The authors may want to explain why infants who died before the age of seven days were excluded from the analysis. Major congenital anomalies, stillbirths etc were already excluded. Why was mortality before seven days also excluded (apart from the congenital diseases)?

The exclusions were made in order to avoid overlapping with our previous publications. Our main interest was in childhood diagnoses which are not given at early neonatal period. This has been clarified in the Methods.

Similarly, a rather large number of missing data on the site of birth is surprising in a country like Finland with high health care standard and excellent registration.

The number of missing data might look high. It is less than 1/1000 cases in the total study sample of 1 053 802 newborns, however.

The data collected included “data on inpatient visits in public hospitals, specialized health care outpatient visits”. It is not clear to me what “… specialized health care outpatient visits” means. Is this general practitioners/health care centres or specialised service like paediatricians, psychiatrists etc? It is also sometimes unclear in the analysis if the data was only collected for hospital visits or also for general health care visits. The difference between hospital visit and hospital admission should be clarified. Was there possibly a different health-care seeking behaviour between the groups?

We have clarified the terms used in the text. Specialized health care outpatient visits include only specialized health care. Visits at general practitioners and health care centers were not included in our study. The comment on possibility of different health-care seeking behavior between the groups is good. In the Discussion we have speculated that such possibility might not be high because of the free public health insurance and general social security system in Finland.

I found the socioeconomic status of “others” somewhat difficult, see comment on results below. The authors may want to explain this. Similarly, combining neurological disorders with behavioural disorders may be questioned – although I can very well see the reason for this. The authors may want to comment on that.

We have combined certain subgroups because of small numbers especially in certain diagnoses.

The study was approved by the Ethics Committee of the Tampere. Obviously, I am not familiar with the process of ethical approvals in Finland - but is ethical approval in Tampere sufficient for a study including the whole of Finland?

The ethical approval in Tampere is valid for whole Finland, which has been added into the text.

The tables are hard to read, quite large and in my version extending over several pages with the header only on the first page. The authors my consider making the tables easier to read, emphasising better what might be significant and having the legends more descriptive.

The tables and the legends have been clarified, as requested.

The first sentence in the results chapter confuses me “Compared to children born in-hospital, the percentage of mothers aged ≤ 19 years was higher in the UOHD group, and mothers aged ≥ 35 was higher in the group of planned home births.” If I read the table correctly, the percentage of mothers aged ≤ 19 years are 1.9% (n=29) in UOHD and in-hospital 2.2%, only a marginal (at best) difference!

We have corrected this, as requested.

When comparing socio-economic status, other or unknown accounts for 40-50% in all groups. That is quite high. The high percentage of unknown is also disturbing, the authors might want to explain that.

As mentioned in the Methods, group “others” includes students, housewives and unclassifiable cases. The data on education and occupation in the health care records are based on self-reporting. Socioeconomic status unknown include mostly of persons, who prefer not to disclose this information. In general it is difficult to define a socio-economic status for young women, since many of them are still in education or at home with child(ren). A clarification on this has been added into the Methods.

In table 3, one line reads “Reimbursement for, n (%)”. Is there something missing?

Missing information has been fulfilled. Reimbursement for medication is correct expression.

Differences in this table are not significant but an ad hoc observation is that POH are younger at first hospital visit, the number of visits is higher, but they are older when reimbursement is registered. This is intriguing.
Dose this only include hospital visits or also primary health care visits?

Both inpatient and outpatient-hospital visits were included as mentioned in the Methods.
The discussion opens with the finding of less infections in the POH and OH groups whereas neurological and mental disorders are more common in the UOH and OH groups. As I mentioned earlier, including the UOH group is somewhat confusing as this probably reflects a different group in terms of maternal background and perinatal complications. The argument that perinatal, maternal, socioeconomic and demographic factors may explain (at least partly) this difference seems logical.

The comparison in this chapter to other countries is difficult, especially with the high standard of care in Finland in mind. The authors compare antibiotic prescription in the perinatal period between Finland and USA. It must be emphasised that these populations are very different. Antibiotic usage in high income countries has been to high (as has been described also in Finland), this problem is probably even more severe in USA than in Finland. In addition, Caesarean section is way more common in USA than in Finland, rendering this comparison of little help. The socioeconomic differences as well as the structure of the health care is also quite different between these countries. Therefore, these comparisons must be made with caution.

Thank you very much for this comment. This led us to seek more information on the use of intra partum antibiotics in Finland. The discussion has been modified according to the new information.

The discussion of the effect on the microbiome is interesting. This theory is becoming more popular – but it must be stressed that this is still a theory. Moreover, the information on perinatal antibiotic treatment in this study was not available.

Yes, the speculation on microbiome is a theory, which has been added into the text.

Finally, as I mentioned before, I find the conclusions drawn by the authors quite strong. The authors mention that the “….. study groups were small, indicating that statistical significances may be difficult to show.” They subsequently add that “….. we realize that our results need to be interpreted with caution.” Subsequently they conclude with the strong statement that the “….risk of childhood morbidity related to asthma and allergic diseases and infection is smaller among children born OH”. I suggest they phrase this conclusion more carefully.

The phrasing has been re-written into a more cautious form.

I also suggest they add to the discussion that a selection bias may certainly account for some of the changes observed as the mothers in the POH group were older, were more often multipara, of upper socioeconomic class, infants were delivered at term, they smoked less etc.

The selection bias has been discussed, as requested.

As mentioned in the beginning, a study on this topic is important. The authors should certainly be acknowledged for this extensive study. Analysing and discussing the data carries huge responsibility and must be interpreted with caution.

Thank you very much for the comments.
