# Supplementary material for: Is birth out-of-hospital associated with mortality and morbidity by seven years of age?
Source: PLoS One. 2021 Apr 21;16(4):e0250163. doi: 10.1371/journal.pone.0250163 (PMC8059817; doi:10.1371/journal.pone.0250163)
Supplement: Attachment — Submitted filename: Response_to_Reviewers18321.docx [file pone.0250163.s003.docx]

Editorial Office

PLOS ONE	March 18, 2021

Please find attached our revised manuscript entitled “Is birth out-of-hospital associated with mortality and morbidity by 7 years of age?” to be considered for publication in PLOS ONE. We appreciate the interest that the editors and reviewers have taken in our manuscript and the constructive criticism and feedback they have given. These changes have clearly improved our manuscript and we would like to thank the reviewers for the constructive criticism and feedback.

PONE-D-20-37796R1
Is birth out-of-hospital associated with mortality and morbidity by 7 years of age?
PLOS ONE

Dear Dr. Ovaskainen,

Thank you for submitting your manuscript to PLOS ONE. After careful consideration, we feel that it has merit but does not fully meet PLOS ONE’s publication criteria as it currently stands. Therefore, we invite you to submit a revised version of the manuscript that addresses the points raised during the review process.

The revised version has considered most of the concerns by reviewers. However, we kindly ask you to address specifically the following issues:

1) While the first reviewer accepted the revised version, and the second reviewer asked for minor revisions, the issues raised reg. the comparability of the study groups, and the conclusions which can be made on this basis are quite fundamental. We ask you therefore to revise the paper by weakening statements, in particular general conclusions in the abstract, results, discussion and conclusions sections about health differences between the two groups born in / outside hospital. (E.g. instead of stating "Results showed that...", write "Results indicated that..." or "Differences appeared to be...").

We have further weakened statements.

2) Please check English language (or use a professional service as support), especially in the modified text, to improve its clarity.

We have used a proofreading service for reviewing this latest revised manuscript.

3) Tables: make sure the title is above each table, and provide a legend for abbreviations and definitions. Table 6, please remove empty rows and columns.

Above mentioned things have revised in tables.

4) In the submission form, question "Financial Disclosure": please state the funding agency (which is now partly named in the manuscript acknowledgements). Provide also a reply reg. competing interests.

This will be corrected in the submission form.

5) In the submission form, you state "all data are available", but tables contain only summary statistics. Explain in the form, section "data availability" why the full dataset cannot be accessed.

All relevant data are within the manuscript and its tables. The original register data with identification codes is confidential and available only for the study group and the holding register worker. The original data received from register holders cannot be shared publicly. This will be added also in the submission form.
